# Dietary branched-chain amino acids intake in relation to general and central obesity among Chinese children and adolescents: a cross-sectional study

**DOI:** 10.3389/fnut.2026.1803824

**Published:** 2026-05-21

**Authors:** Yao Chen, Hongwei Wang, Changqing Liu, Yiya Liu, Qianrang Zhu, Meina Tian, Zhenchuang Tang, Lianlong Yu

**Affiliations:** 1 Department of Clinical Nutrition, People's Hospital of Rizhao, Rizhao, China; 2 Department of Health Care, People's Hospital of Rizhao, Rizhao, China; 3 Hebei Center for Disease Control and Prevention, Shijiazhuang, China; 4 Guizhou Center for Disease Control and Prevention, Guiyang, China; 5 Jiangsu Provincial Center for Disease Control and Prevention, Nanjing, China; 6 Institute of Food and Nutrition Development, Ministry of Agriculture and Rural Affairs, Beijing, China; 7 School of Public Health, Shandong Second Medical University, Weifang, China; 8 Shandong Center for Disease Control and Prevention, Jinan, China; 9 School of Public Health, Shandong First Medical University, Jinan, China

**Keywords:** children, adolescents, branched-chain amino acids, central obesity, general obesity, machine learning

## Abstract

**Background:**

Epidemiological evidence on dietary branched-chain amino acids (BCAAs) and obesity remain inconsistent, particularly for Chinese children and adolescents. This study investigated their association using nationally representative data.

**Methods:**

Data were obtained from the 2016–2019 China Children and Lactating Women Nutrition and Health Surveillance, which contained 12,542 school-aged participants. Dietary BCAAs intake was determined with a food-frequency questionnaire supplemented with a weighed condiment inventory and expressed as energy density (g/1,000 kcal). General and central obesity were defined according to body mass index and waist circumference criteria, respectively. Multivariable logistic regression and stratified analyses were utilized to estimate associations. Restricted cubic spline (RCS) analyses were utilized to investigate possible dose-response relationships. In addition, machine learning (ML) models were used to assess the predictive performance and the relative importance of individual features.

**Results:**

After adjustment for potential confounders, higher BCAAs intake was associated with increased risks of both general and central obesity. Compared with the lowest tertile (T1), the highest tertile (T3) had significantly higher risks of general obesity (OR = 1.52, 95% CI: 1.31–1.76) and central obesity (OR = 1.37, 95% CI: 1.19–1.56). Each standard deviation increase in BCAAs intake was linked to a 12% greater risk of general obesity and a 9% greater risk of central obesity. RCS models showed a significant non-linear, positive dose-response relationship between BCAAs intake and obesity risk. Subgroup analyses revealed a consistent positive association between BCAAs and general obesity across all subgroups. For central obesity, a stronger association was specifically observed in males. ML analyses identified leucine as the most influential BCAAs component for predicting obesity risk.

**Conclusion:**

Increased dietary intake of BCAAs is associated with higher risks of both general and central obesity among Chinese children and adolescents, with leucine showing the strongest association among the three BCAAs.

## 1 Introduction

The rising occurrence of obesity and overweight in adolescents and children is now a key public health problem worldwide (1). In China, the proportion of adolescents and children suffering from overweight and obesity is still steadily increasing (2, 3). These conditions not only make the early-onset metabolic disorders such as abnormalities in lipid and glucose metabolism and cardiovascular diseases more likely, but also impair the psychological development, social functioning, and overall quality of life in this population (4, 5). Although dietary imbalance is generally accepted as a major contributor to excessive weight gain, most of the research to date has centered on macronutrient intake or dietary patterns in general. Consequently, the potential roles of certain micronutrients, especially branched-chain amino acids (BCAAs) in the advancement of overweight and obesity are not sufficiently investigated in a systematic manner (6).

Mechanistically, BCAAs are nutritionally indispensable amino acids implicated in protein synthesis, muscle maintenance, and hormonal regulation; they may affect body-weight homeostasis via a number of biological pathways (7, 8). Despite this biological plausibility, epidemiological studies examining this link have been inconsistent regarding dietary BCAAs intake and obesity in adult populations. Studies conducted outside China have reported mixed findings, with some showing positive associations (9–11) and others reporting inverse (12, 13). Within Chinese adults, results have also been conflicting: national surveys have demonstrated positive associations between BCAAs intake and obesity (14, 15), whereas a study in northern Chinese young adults reported an inverse relationship (16).

Of note, contrary to the inconsistent results in the adult population, the limited data available in adolescents and children more consistently suggest a positive association between dietary BCAAs intake and risk of obesity. For instance, one study of adolescents (12–18 years of age) in the United States found significant positive links between BCAAs consumption, BMI, and total body fat percentage (17). Likewise, a prospective follow-up study of offspring born to mothers with gestational diabetes mellitus in China showed that raised dietary BCAAs intake among children was substantially linked to raised risk of childhood overweight and obesity (18).

Thus, existing studies in children point toward a positive association. Although these observations suggest the potentially detrimental role of BCAAs in the pathogenesis of childhood obesity, most epidemiological studies to date have focused on adult or elderly populations. Large-scale and systematic studies in adolescents and children specifically are still scarce, especially well-designed research that is based on Chinese cohorts. Because adolescence and childhood are crucial periods for the emergence and prevention of obesity, there is a critical need for comprehensive evaluations using nationally representative data sets.

To bridge this knowledge gap, the present investigation is based on data from the China children and lactating women nutrition and health surveillance (CCLWNHS) to examine the link between dietary BCAAs intake and the risk of obesity in Chinese adolescents and children. The findings are expected to provide strong evidence for targeted nutritional interventions to prevent childhood obesity and to support the advancement of effective public health approaches and policies.

## 2 Methods

### 2.1 Study design and participants

The relevant dataset was derived from the 2016–2019 CCLWNHS, a nationally organized program conducted by the National Institute for Nutrition and Health of the Chinese Center for Disease Control and Prevention (China CDC). A multistage, stratified, cluster random sampling approach was applied to recruit representative participants from four provinces, Jiangsu, Hebei, Shandong, and Guizhou, corresponding to southern, eastern, northern, and western regions of China, respectively. These provinces represent a wide variety of socioeconomic conditions and dietary cultures, and thus allow the capture of regional variability in dietary behaviors and nutritional status among adolescents and children. All the enrolled participants underwent standardized face-to-face interviews, anthropometric measurements, laboratory exams, and dietary surveys. Data collection was carried out following uniform protocols and using calibrated instruments to ensure consistency, accuracy and reliability (19). The study protocol received ethical approval from the Ethics Review Committee of the China CDC (approval number: 201614), and written informed consent was obtained from all participants and their legal guardians.

A total of 12,976 adolescents and children were recruited initially ranging from 6–18 years. Participants were then excluded stepwise, if they met any of the following criteria: age outside the target range (*n* = 20), missing or implausible BMI values (*n* = 5), missing or extreme WC measurements (*n* = 21), incomplete dietary information (*n* = 124), or implausible total energy intake, which was defined as values within the lowest or highest 1% after stratification by age and sex (*n* = 264). After these exclusions, 12,542 participants remained and formed the final analytical sample.

### 2.2 Dietary evaluation

Dietary information was obtained by certified professionals with face-to-face interview using a food frequency questionnaire (FFQ) developed and rigorously validated by experts at the China CDC for reliability and accuracy. The FFQ is a record of both the frequency and portion size of food items in a wide range and has been widely used in large scale nutritional investigations in Chinese young individuals (20–23). Investigators used standardized visual aids and measuring tools to enhance the accuracy of food frequency and portion estimates. For younger subjects or subjects not capable of completing the questionnaire independently, information was gathered with the assistance of parents or guardians. To enhance the precision of dietary assessment, dietary intake of condiments was further measured using a consecutive 3-day weighed inventory method. This approach captured household and school canteen use of cooking oil, salt, monosodium glutamate and other commonly used seasonings. Individual nutrient and amino acid intakes were then determined based on a standardized food composition database (24), which offers an extensive and uniform estimation of dietary exposure.

### 2.3 Ascertainment of obesity

Anthropometric measurements were done according to the Chinese industry standard Human Health Monitoring: Anthropometric Methods (WS/T 424–2013) (25), and the instruments satisfied the national metrological certification requirements. Weight status was categorized as normal weight, overweight, and obesity based on sex- and age-specific BMI cut-off values defined by the Chinese national health industry standard Screening for Overweight and Obesity among School-age Adolescents and children (WS/T 586–2018) (26). Central obesity was defined by WC percentile values (75th and 90th percentiles) according to the standard Screening Thresholds for High WC among Adolescents and children Aged 7–18 Years (WS/T 611–2018) (27), and additional reference values from a national survey among Chinese preschool children aged 3–7 years (28). On this basis, participants were further divided into normal, pre-abdominal obesity and abdominal obesity groups.

### 2.4 Covariates

Several potential confounders were considered to adjust for covariates that may affect the link between dietary BCAAs intake and obesity outcomes. These covariates included sociodemographic characteristics, physical activity indicators, macronutrient intake variables. Sociodemographic factors included sex, age, and school level (primary school/secondary school). Physical activity was evaluated according to the achievement of the recommended level of moderate-to-vigorous physical activity (MVPA: yes/no). Although MVPA duration was also collected, it showed high collinearity with the binary MVPA indicator. Thus, only the categorical variable indicating compliance with MVPA recommendations was kept in the multivariable models to prevent instability, which is a consequence of multicollinearity. Dietary covariates comprised daily intakes of protein, fat and carbohydrates (g/day) to adjust for overall macronutrient consumption. Information on sex, age, outdoor activity behaviors and duration of activity were collected using self-reported questionnaires during the survey.

### 2.5 Machine learning algorithms

Extreme Gradient Boosting (XGBoost) is an ensemble learning algorithm that improves predictive accuracy by building decision trees. Every new tree tries to fix the mistakes of its predecessors and the combination of several trees significantly increases the overall prediction accuracy. XGBoost has inbuilt features to avoid overfitting in training and can automatically estimate the significance of each variable, which is why it is popular in medical risk prediction (29, 30).

Light Gradient Boosting Machine (LightGBM) is a gradient boosting model that is tailored to work with large-scale data. It greatly lowers the computational cost by disregarding a part of samples with small gradients and combining correlated features. LightGBM, in contrast to the traditional level-based growth strategies, biases the expansion of branches with higher prediction errors, leading to a faster convergence and reduced memory usage. LightGBM is effective in controlling computation time in large sample sizes like those in the current study, which has more than 10,000 samples (31, 32).

Naïve Bayes (NB) is a Bayesian classification algorithm. Its fundamental concept is to estimate the type of a new sample based on the probability distribution of each type based on past data. NB is easy to code and computationally inexpensive. NB has good computational efficiency even in cases where the number of features is relatively large, and thus is frequently used as a baseline model to establish whether more complex algorithms provide real benefits (33, 34).

Neural Networks (NN) is a computational model that simulates biological neurons by using multi-layer weighted computations and non-linear transformations, gradually deriving higher-level features to produce predictions. It is able to capture complex interactions between variables but cannot be easily interpreted and needs large datasets to be trained. NN in this study was applied to investigate the maximum number of features that can be combined and to compare the performance of the NN with tree-based models (35, 36).

### 2.6 Statistical analyses

Because dietary BCAAs intake was highly correlated with total energy intake, the BCAAs exposure was adjusted using the energy density approach, as recommended in previous methodological studies (14, 37). Accordingly, BCAAs consumption was normalized and expressed in grams per 1,000 kilocalories (g/1,000 kcal). This normalization helps minimize confounding due to total energy intake, helps reduce the risk of multicollinearity in regression models and helps to better represent the proportional contribution of BCAAs in the total dietary composition, allowing the association between BCAAs intake patterns and obesity to be evaluated independent of total caloric intake.

Obesity status was defined separately based on two anthropometric parameters (BMI and WC), with each divided into three categories. To ensure comparability between analyses, participants with both normal BMI and normal WC were used as the common reference group in obesity-related subgroup analyses. Because subgroup structures varied, one unified categorization scheme was not applied to the entire sample. Instead, participants were then stratified into tertiles (T1–T3) based on the cutoff points derived from the tertile distribution of BCAAs intake in this reference group, thereby enhancing the comparability of the results.

With the large sample size and the assumptions of the central limit theorem, the sampling distributions of continuous variables were thought to be approximately normal. Accordingly, continuous variables are represented as mean (SD) and categorical variables as counts and percentages. Continuous variables were compared using one-way ANOVA, while categorical variables were analyzed with the chi-square test. Violin plots were created to show differences between the distribution of dietary BCAAs intake between obesity categories. Spearman's rank correlation coefficients were calculated to estimate associations between BCAAs intake and obesity-related indicators and the correlation matrix was visualized using a heatmap. Binary logistic regression models were used to calculate the association between dietary BCAAs intake and obesity under both definitions, with effect estimates represented as odds ratios (ORs) and 95% confidence intervals (CIs). BCAAs intake was modeled both categorically (tertiles, with T1 as the reference group) and continuously (per one SD increment). Three sequential models were specified: crude model with no adjustment of the covariates; Model 1 with adjustment for sex, age, school type, and MVPA status; and Model 2 with an additional adjustment for daily intakes of protein, fat, and carbohydrate. To assess the possibility of effect modification, stratified analyses were conducted based on sex, age, school level, and MVPA status and multiplicative interaction terms were tested. Furthermore, restricted cubic spline (RCS) functions were utilized to examine potential non-linear dose-response relationships between dietary BCAAs intake and risks of general and central obesity. In these models, intake of BCAAs was considered as a continuous variable with 3 knots placed at the 10th, 50th, and 90th percentiles, and median intake was used as the reference value. RCS models were adjusted for sex, age, school type, MVPA, and macronutrient intake (protein, fat, and carbohydrates).

To further assess the predictive power of dietary BCAAs intake to obesity, four machine learning (ML) algorithms were utilized: LightGBM, NN, NB, and XGBoost. Input features included the three individual BCAAs (Isoleucine [Ile], Leucine [Leu], Valine [Val]), sex, age, MVPA status, school type, and daily dietary intakes of protein, fat and carbohydrates, resulting in 10 predictor variables. Model performance was assessed based on the receiver operating characteristic (ROC) curves and the respective area under the curve (AUC), using 10-fold cross-validation to ensure generalizability and robustness. The model with the greatest predictive accuracy was used for further analysis. Shapley additive explanations (SHAP) were used to measure and rank the contribution of each input feature to determine which predictors of obesity risk are most influential. All statistical analyses (including ML procedures) were conducted utilizing R software (version 4.3.1). Statistical significance was defined as *p* < 0.05 using two-sided tests.

## 3 Results

### 3.1 Characteristics of participants

**Figure 1:**
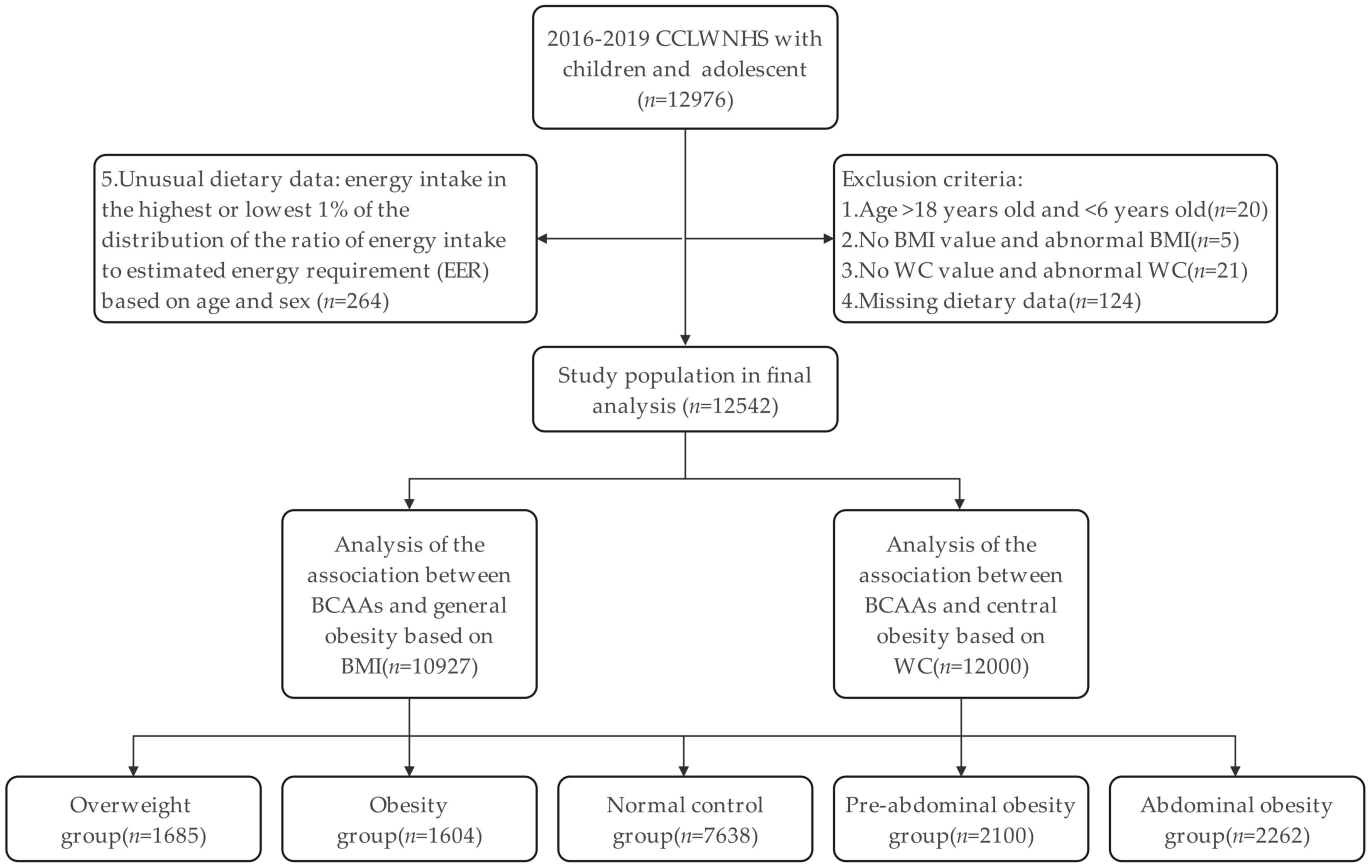
Study flow chart.

**Figure 2:**
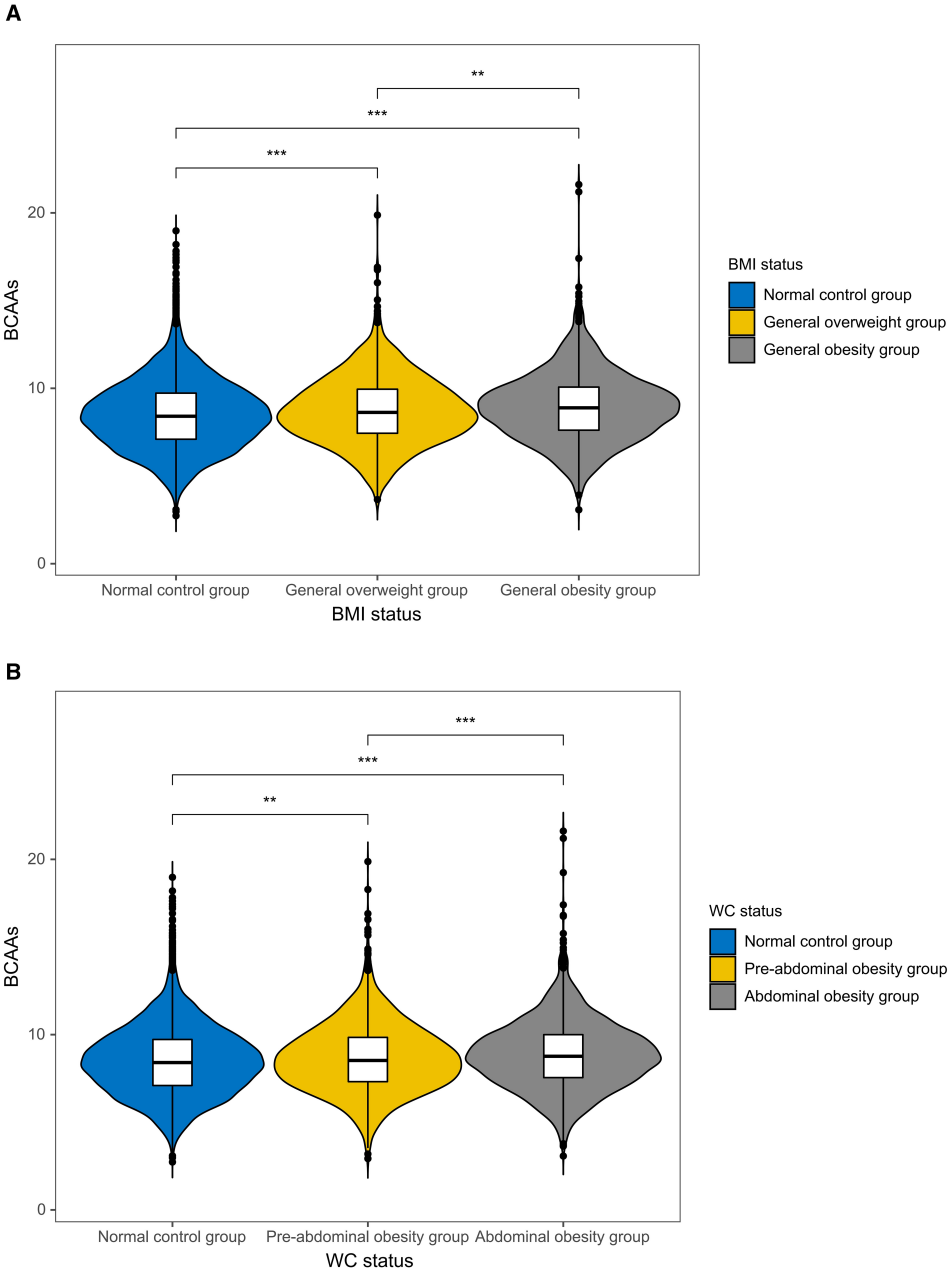
Comparison of dietary BCAAs intake among different obesity status groups. Violin plots showing dietary BCAAs intake in **(A)** general obesity groups (normal, overweight and obesity, based on BMI) and **(B)** central obesity groups (normal, pre-abdominal obesity, and abdominal obesity, based on WC). Asterisks show statistically substantial variances between groups (**p < 0.01; ***p < 0.001).

A total of 12,976 Chinese adolescents and children were first included in the study. After the predefined inclusion and exclusion criteria, 12,542 participants remained in the final analytical sample (Figure 1). Participants were divided into tertiles of dietary BCAAs intake low (T1), medium (T2) and high (T3). As indicated in Table 1, there were substantial variances between the three intake groups with regard to demographic characteristics, anthropometric measurements, metabolic risk factors, and nutrient intake. Relative to those in the lowest tertile (T1), those in higher BCAAs intake groups tended to be younger, had a higher proportion of females and a higher MVPA participation (*p* < 0.01). Increased BCAAs intake was also linked to raised prevalence of both general and central obesity (*p* < 0.01). Figure 2 shows a stepwise increase in dietary BCAAs intake across obesity categories, consistent for both general and central obesity.

**Table 1:** Participant characteristics according to tertiles of dietary BCAAs intake.

Parameters	Overall	Tertiles of dietary BCAAs intake (g/1,000 kcal)			*p*-*value*
		T1 (<7.569)	T2 (7.569–9.239)	T3 (>9.239)	
Anthropometric and characteristics	
Male, *n* (%)	6,260 (49.91)	2,066 (52.99)	2,093 (49.48)	2,101 (47.61)	<0.001
Age, years	11.22 (3.21)	11.67 (3.20)	11.15 (3.16)	10.90 (3.22)	<0.001
School type, *n* (%)					<0.001
Primary school	7,525 (60.00)	2,132 (54.68)	2,593 (61.30)	2,800 (63.45)	
Secondary school	5,017 (40.00)	1,767 (45.32)	1,637 (38.70)	1,613 (36.55)	
MVPA, *n* (%)	9,917 (79.07)	2,978 (76.38)	3,445 (81.44)	3,494 (79.18)	<0.001
MVPA duration, min	46.79 (47.96)	46.40 (52.35)	47.86 (47.54)	46.11 (44.15)	0.196
BMI, kg/m^2^	18.89 (3.98)	18.77 (3.85)	18.89 (3.96)	19.00 (4.09)	0.036
WC, cm	64.11 (11.08)	63.92 (10.97)	64.17 (10.91)	64.21 (11.32)	0.455
WHtR	0.43 (0.06)	0.43 (0.05)	0.43 (0.06)	0.43 (0.06)	0.040
General obesity, *n* (%)					<0.001
Normal	7,638 (69.90)	2,546 (74.90)	2,546 (69.43)	2,546 (65.94)	
Overweight	1,685 (15.42)	466 (13.71)	587 (16.01)	632 (16.37)	
Obesity	1,604 (14.68)	387 (11.39)	534 (14.56)	683 (17.69)	
Central obesity, *n* (%)					<0.001
Normal	7,638 (63.65)	2,546 (67.91)	2,546 (62.82)	2,546 (60.65)	
Pre-abdominal obesity	2,100 (17.50)	631 (16.83)	726 (17.91)	743 (17.70)	
Abdominal obesity	2,262 (18.85)	572 (15.26)	781 (19.27)	909 (21.65)	
Metabolic risk index	
TyG	8.13 (0.41)	8.11 (0.40)	8.13 (0.42)	8.13 (0.41)	0.076
METS-IR	26.25 (6.54)	26.19 (6.39)	26.23 (6.52)	26.32 (6.69)	0.621
TG/HDL	1.28 (0.88)	1.30 (0.86)	1.29 (0.94)	1.26 (0.84)	0.089
VAI	2.11 (1.53)	2.14 (1.51)	2.12 (1.64)	2.07 (1.45)	0.123
METS-VF	4.35 (1.00)	4.36 (1.03)	4.35 (1.00)	4.33 (0.99)	0.283
ABSI	0.07 (0.01)	0.07 (0.01)	0.07 (0.01)	0.07 (0.01)	0.238
BRI	2.26 (0.96)	2.22 (0.92)	2.27 (0.96)	2.28 (1.00)	0.020
CMI	0.68 (0.52)	0.69 (0.53)	0.68 (0.54)	0.66 (0.48)	0.022
AIP	1.76 (0.22)	1.78 (0.22)	1.76 (0.23)	1.75 (0.22)	<0.001
Laboratory measurements	
Hemoglobin, g/L	137.65 (13.09)	138.02 (13.62)	137.34 (13.31)	137.62 (12.38)	0.062
FBG, mmol/L	5.07 (0.58)	4.96 (0.58)	5.09 (0.59)	5.14 (0.56)	<0.001
TC, mmol/L	3.87 (0.72)	3.75 (0.68)	3.88 (0.72)	3.96 (0.74)	<0.001
TG, mmol/L	0.91 (0.42)	0.92 (0.41)	0.91 (0.43)	0.91 (0.41)	0.512
HDL-C, mmol/L	1.50 (0.35)	1.46 (0.34)	1.51 (0.36)	1.53 (0.35)	<0.001
LDL-C, mmol/L	2.10 (0.61)	2.03 (0.58)	2.10 (0.61)	2.16 (0.64)	<0.001
Uric acid, μmol/L	313.70 (82.62)	319.91 (83.16)	311.45 (82.37)	310.39 (82.08)	<0.001
Creatinine, μmol/L	51.83 (13.54)	53.17 (14.67)	51.49 (12.91)	50.96 (12.99)	<0.001
Ferritin, μg/L	68.43 (46.72)	69.24 (46.75)	67.25 (45.54)	68.86 (47.80)	0.119
Transferrin, g/L	3.52 (1.26)	3.60 (1.36)	3.50 (1.25)	3.48 (1.18)	<0.001
Albumin, g/L	49.17 (2.93)	49.21 (2.92)	49.15 (2.97)	49.15 (2.90)	0.581
Nutritional parameters	
Ile, g/1,000 kcal	1.97 (0.42)	1.55 (0.19)	1.93 (0.14)	2.39 (0.33)	<0.001
Leu, g/1,000 kcal	3.97 (0.94)	2.94 (0.45)	3.90 (0.24)	4.95 (0.60)	<0.001
Val, g/1,000 kcal	2.64 (0.65)	1.93 (0.28)	2.58 (0.18)	3.33 (0.42)	<0.001
BCAAs, g/1,000 kcal	8.59 (1.97)	6.42 (0.88)	8.41 (0.47)	10.66 (1.29)	<0.001
Protein, g/day	120.75 (75.84)	94.12 (57.07)	119.36 (63.81)	145.62 (91.22)	<0.001
Fat, g/day	45.20 (42.40)	44.84 (49.65)	46.06 (39.62)	44.70 (37.77)	0.267
Carbohydrate, g/day	351.25 (199.55)	400.37 (230.75)	349.61 (178.35)	309.43 (178.24)	<0.001
Total energy, kcal/day	2,294.81 (1,316.69)	2,381.49 (1,426.35)	2,290.38 (1,220.33)	2,222.46 (1,300.43)	<0.001

Mean (SD) was used for continuous measures and number (%) for categorical indicators.

WHtR, Waist-to-height ratio; TyG, Triglyceride-glucose index; TG/HDL, Triglycerides/High-density lipoprotein index; METS-IR, Metabolic score for insulin resistance; VAI, Visceral adiposity index; ABSI, A body shape index; METS-VF, Metabolic score for visceral fat; BRI, Body roundness index; CMI, Cardiometabolic index; AIP, Atherogenic index of plasma.

Cutoff values of BCAAs tertiles are as follows: T1: <7.569g/1,000 kcal; T2: 7.569–9.239g/1,000 kcal; T3: >9.239g/1,000 kcal.

**Figure 3:**
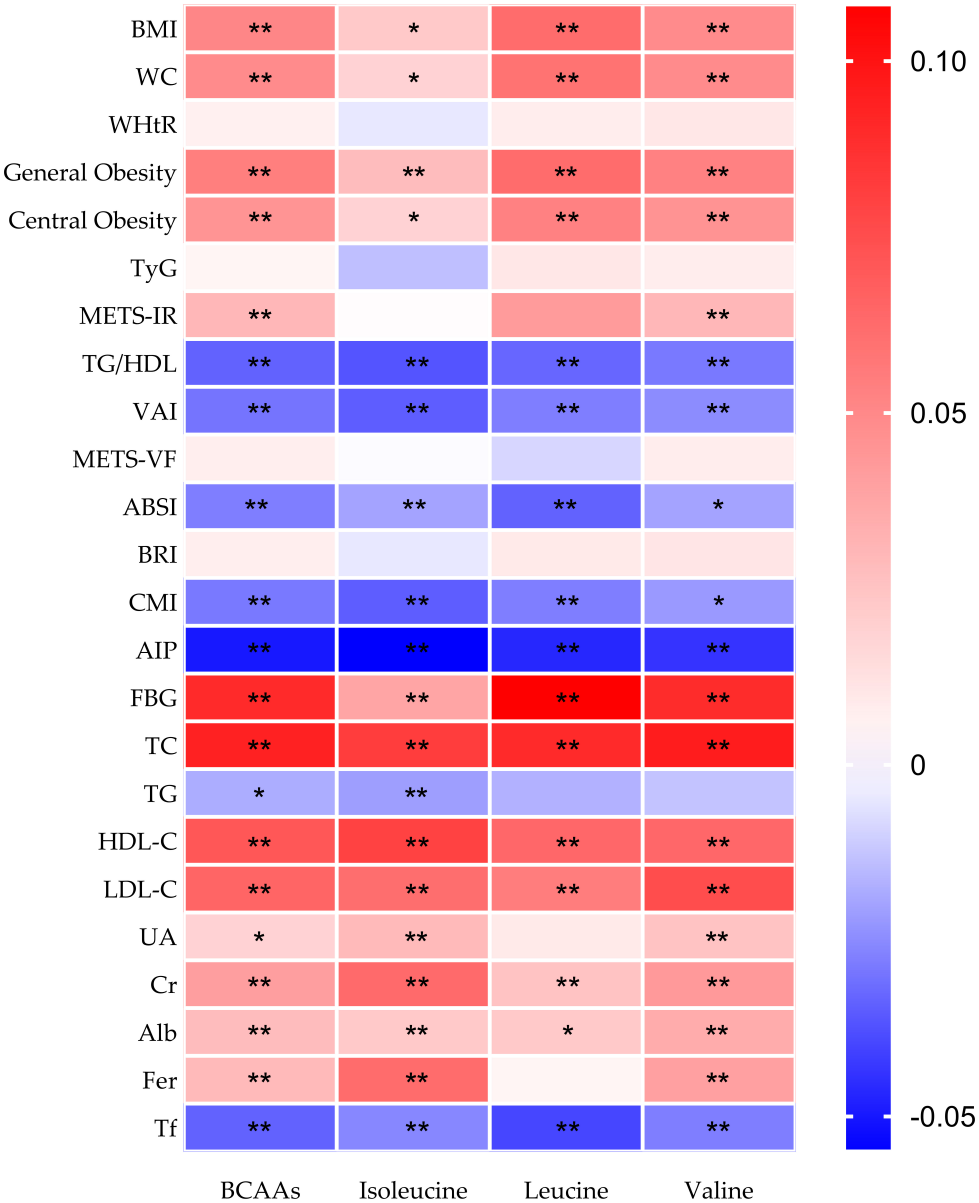
Heatmap of correlations between BCAAs and metabolic, anthropometric, and biochemical indicators. The color gradient shows the direction and strength of correlations, with red indicating positive associations, and blue indicating negative associations. Statistical significance is represented by an asterisk (*) for *p* < 0.05 and a double asterisk (**) for *p* < 0.01. WHtR, Waist-to-Height Ratio; TyG, Triglyceride-Glucose Index; TG/HDL, Triglycerides/High-Density Lipoprotein Index; METS-IR, Metabolic Score for Insulin Resistance; VAI, Visceral Adiposity Index; ABSI, A Body Shape Index; METS-VF, Metabolic Score for Visceral Fat; BRI, Body Roundness Index; CMI, Cardiometabolic Index.

Figure 3 shows a correlation heatmap of associations between total BCAAs intake, its individual components (Ile, Leu, and Val) and indicators of obesity. Total and individual BCAAs were significantly and positively linked with anthropometric parameters, such as BMI, WC, general obesity and central obesity, and selected biochemical parameters, such as TC, FBG, LDL-C, and Cr (all *p* < 0.05).

### 3.2 Association between dietary BCAAs and obesity

In multivariable-adjusted models, participants in the highest BCAAs intake tertile had significantly higher risks of general obesity (OR = 1.52, 95% CI: 1.31–1.76) and central obesity (OR = 1.37, 95% CI: 1.19–1.56) compared with the lowest tertile. Each standard deviation (SD) increment in BCAAs intake was associated with a 12% higher risk of general obesity and a 9% higher risk of central obesity (Tables 2, 3). Further analyses showed that all three individual BCAAs, Ile, Leu, and Val, were independently and positively related with both general and central obesity (see Supplementary Tables S1–S6).

**Table 2:** ORs and corresponding 95% CIs according to dietary BCAAs intake and the risk of general obesity.

Variable	Classified analysis			Continuous analysis		
	T1	T2	T3	*p* for trend	Per 1-SD	*p*-value
Overweight	
Case/control subjects, *n*	466/2,546	587/2,546	632/2,546		1,685/7,638	
Crude model	1	1.26 (1.10–1.44)	1.36 (1.19–1.55)	<0.001	1.07 (1.04–1.10)	<0.001
Model 1	1	1.28 (1.12–1.47)	1.39 (1.22–1.59)	<0.001	1.07 (1.04–1.10)	<0.001
Model 2	1	1.27 (1.09–1.48)	1.38 (1.14–1.66)	0.001	1.09 (1.04–1.14)	<0.001
Obesity	
Case/control subjects, *n*	387/2,546	534/2,546	683/2,546		1,604/7,638	
Crude model	1	1.38 (1.20–1.59)	1.77 (1.54–2.02)	<0.001	1.12 (1.09–1.15)	<0.001
Model 1	1	1.38 (1.20–1.59)	1.77 (1.54–2.03)	<0.001	1.12 (1.09–1.15)	<0.001
Model 2	1	1.35 (1.15–1.59)	1.71 (1.40–2.08)	<0.001	1.14 (1.09–1.20)	<0.001
General obesity	
Case/control subjects, *n*	853/2,546	1,121/2,546	1,315/2,546		3,289/7,638	
Crude model	1	1.31 (1.18–1.46)	1.54 (1.39–1.71)	<0.001	1.09 (1.07–1.12)	<0.001
Model 1	1	1.33 (1.19–1.48)	1.56 (1.40–1.76)	<0.001	1.10 (1.07–1.12)	<0.001
Model 2	1	1.31 (1.16–1.47)	1.52 (1.31–1.76)	<0.001	1.12 (1.07–1.16)	<0.001

Model 1: Adjusted for age (continuous), sex (female or male), MVPA (yes or no), school type (primary school or secondary school). Model 2: Further adjusted for daily intake of protein, fat, and carbohydrates (all continuous). SD, standard deviation.

**Table 3:** ORs and corresponding 95% CIs according to dietary BCAAs intake and the risk of central obesity.

Variable	Classified analysis			Continuous analysis		
	T1	T2	T3	*p* for trend	Per 1-SD	*p-value*
Pre-abdominal obesity	
Case/control subjects, *n*	631/2,546	726/2,546	743/2,546		2,100/7,638	
Crude model	1	1.15 (1.02–1.30)	1.18 (1.05–1.33)	0.008	1.04 (1.02–1.07)	0.001
Model 1	1	1.16 (1.03–1.31)	1.18 (1.05–1.33)	0.007	1.04 (1.02–1.07)	0.001
Model 2	1	1.17 (1.02–1.34)	1.20 (1.01–1.42)	0.046	1.07 (1.03–1.12)	0.001
Abdominal obesity	
Case/control subjects, *n*	572/2,546	781/2,546	909/2,546		2,262/7,638	
Crude model	1	1.37 (1.21–1.54)	1.59 (1.41–1.79)	<0.001	1.10 (1.07–1.12)	<0.001
Model 1	1	1.39 (1.23–1.57)	1.63 (1.44–1.83)	<0.001	1.10 (1.07–1.13)	<0.001
Model 2	1	1.36 (1.19–1.56)	1.56 (1.32–1.85)	<0.001	1.11 (1.06–1.15)	<0.001
Central obesity	
Case/control subjects, *n*	1,203/2,546	1,507/2,546	1,652/2,546		4,362/7,638	
Crude model	1	1.25 (1.14–1.38)	1.37 (1.25–1.51)	<0.001	1.07 (1.05–1.09)	<0.001
Model 1	1	1.27 (1.16–1.40)	1.40 (1.27–1.53)	<0.001	1.07 (1.05–1.09)	<0.001
Model 2	1	1.26 (1.13–1.40)	1.37 (1.19–1.56)	<0.001	1.09 (1.06–1.13)	<0.001

Model 1 was adjusted for age (continuous), sex (female or male), MVPA (yes or no), school type (primary school or secondary school). Model 2 was further adjusted for protein (continuous), fat (continuous), carbohydrate (continuous).

SD, standard deviation.

**Figure 4:**
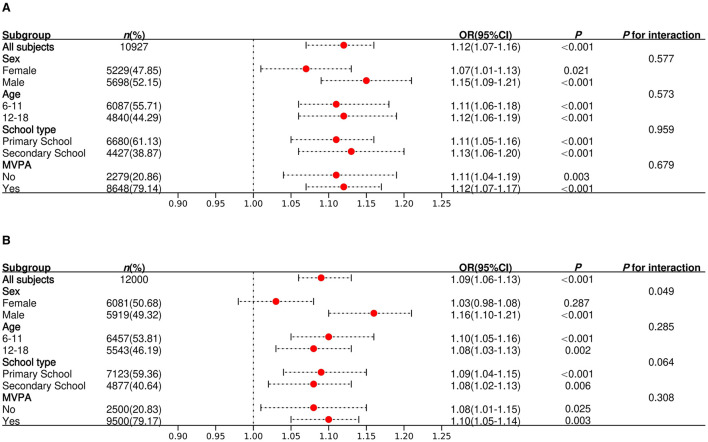
Stratified analysis of the association of dietary BCAAs intake with risk of obesity. Forest plots show multivariable-adjusted ORs and 95% CIs for the association per 1-SD increase in BCAAs intake for subgroups. **(A)** Association with general obesity (BMI-defined). **(B)** Association with central obesity (WC-defined). Regression models were adjusted for age (continuous), sex (female or male), MVPA (yes or no), school type (primary school or secondary school), and daily intake of protein, fat and carbohydrates (all continuous). *p* for interaction values indicate whether the effect of BCAAs intake on the risk of obesity differs by subgroup. The *p* for interaction values test for effect modification by each of the subgroup variables.

The results of stratified analyses by sex, age, school type and level of MVPA participation are presented in Figure 4. The positive link between dietary BCAAs intake and general obesity risk was statistically significant in all subgroups (all *p* < 0.05), and no significant interactions were found (*p* for interaction > 0.05). However, a significant sex interaction was observed for central obesity, with a stronger positive association in males than in females (*p* for interaction = 0.049).

**Figure 5:**
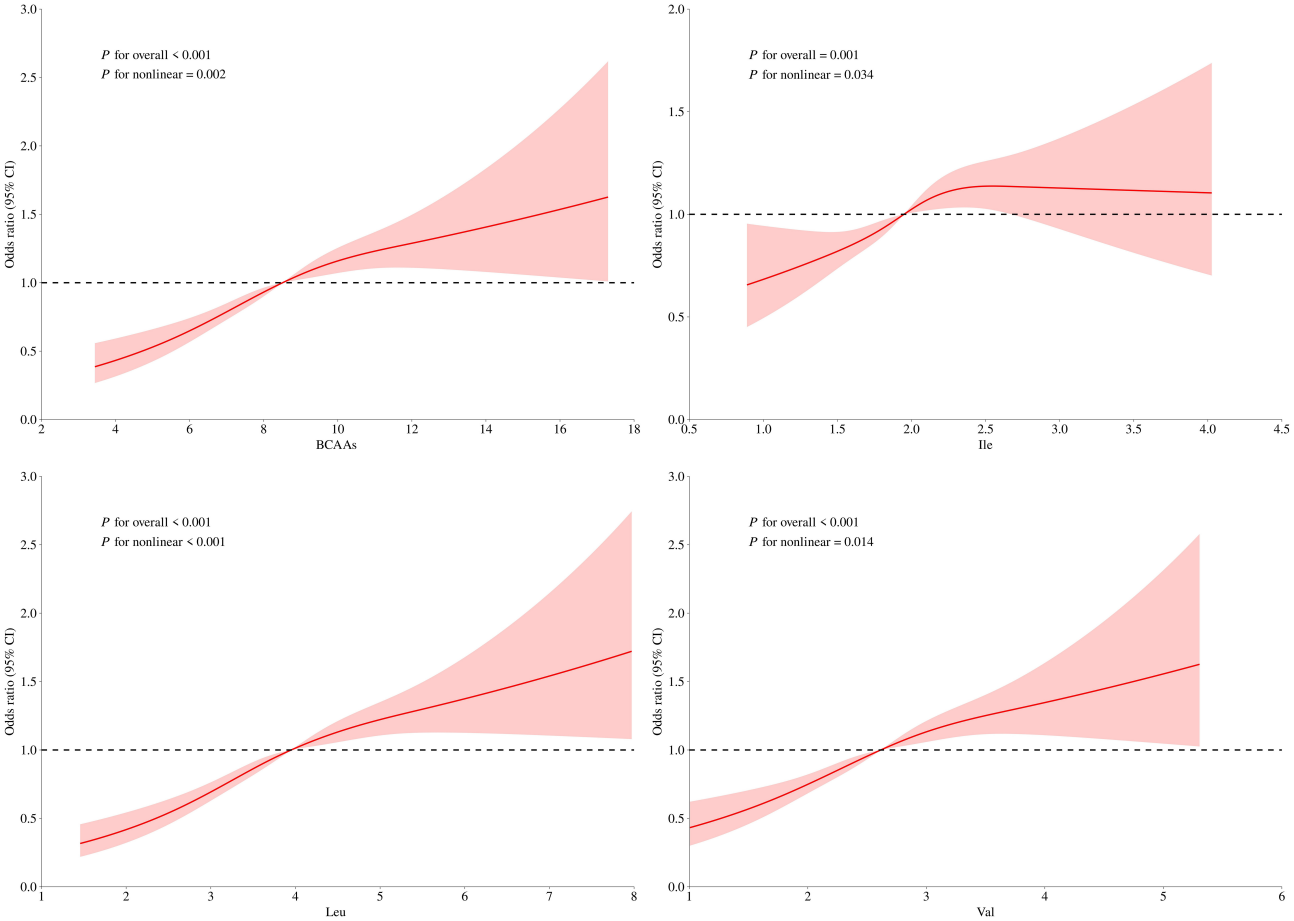
Non-linear relationships between dietary BCAAs intake and general obesity risk. Curves represent multivariable-adjusted ORs (solid lines) and 95% CIs (shaded areas) for the relationship of **(A)** total BCAAs, **(B)** Ile, **(C)** Leu, and **(D)** Val intake with BMI-defined general obesity. Models were adjusted for age (continuous), sex (female or male), MVPA (yes or no), school type (primary school or secondary school), and daily intake of protein, fat and carbohydrates (all continuous).

**Figure 6:**
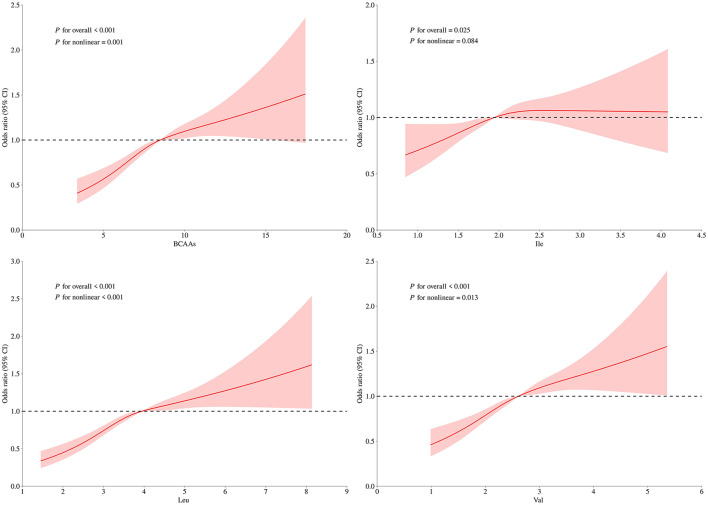
Non-linear relationship between dietary BCAAs intake and central obesity risk. Curves represent multivariable-adjusted ORs (solid lines) and 95% CIs (shaded areas) for the relationship of **(A)** total BCAAs, **(B)** Ile, **(C)** Leu, and **(D)** Val intake with WC-defined central obesity. Models were adjusted for sex (male or female), age (continuous), MVPA (yes or no), school type (primary school or secondary school) and daily intake of protein, fat, and carbohydrates (all continuous).

Figures 5 and 6, which were generated from RCS models, showed significant non-linear positive associations between dietary BCAAs intake and the risks of both general and central obesity (*p* < 0.05). The risk of obesity was progressively higher with increasing BCAAs intake, but the association tended to level off at the upper intake range, indicating a possible saturation effect or metabolic threshold. Similar non-linear trends were found for Leu and Val, while the relationship between Ile and central obesity was found to be roughly linear.

### 3.3 Ranking of BCAAs components based on ML models

**Figure 7:**
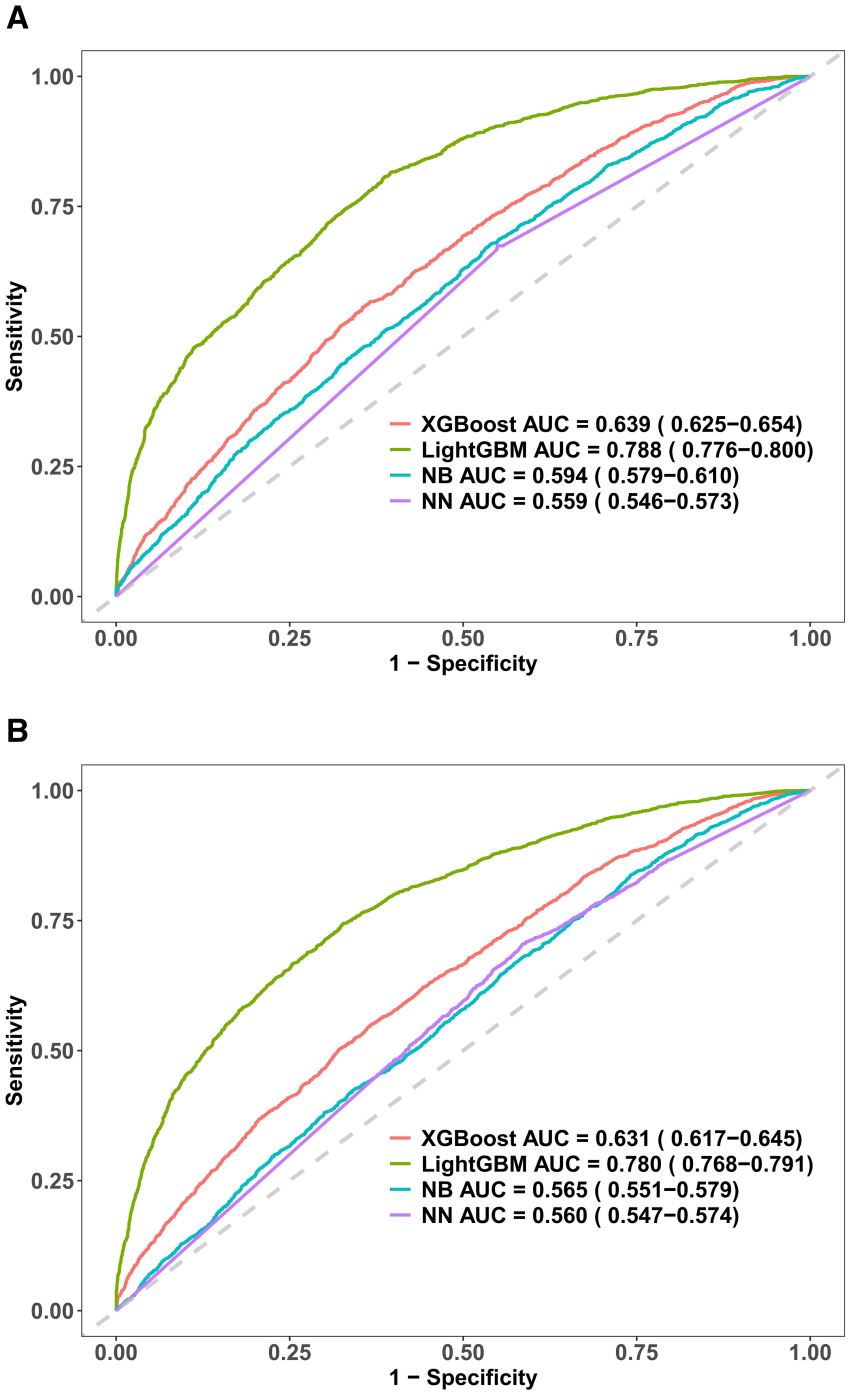
ROC curves for four ML models for predicting obesity. ROC curves are presented for **(A)** general obesity (based on BMI) and **(B)** central obesity (based on WC) with the AUC values and 95% CI for each model: XGBoost, LightGBM, NB, and NN.

**Figure 8:**
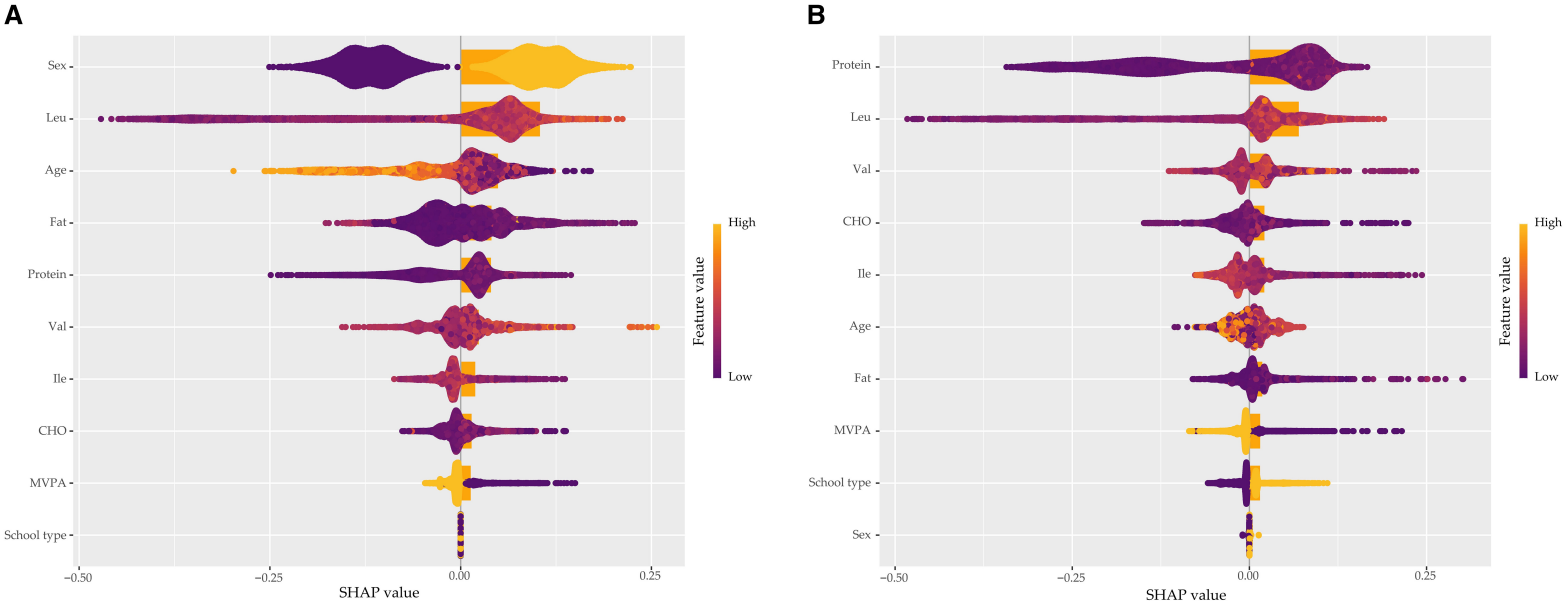
Feature importance interpretation for the LightGBM model using SHAP values. SHAP values show the impact of each feature to the prediction of **(A)** general obesity and **(B)** central obesity. Features are ranked by importance with positive SHAP values indicating increased risk of obesity and negative values indicating protective effects.

Figure 7 illustrates the performance of four ML algorithms in the prediction of obesity using ROC curves. Among the models, LightGBM showed the best predictive power to identify both general and central obesity with AUC scores of 0.788 (95% CI: 0.776–0.800) and 0.780 (95% CI: 0.768–0.791) for general and central obesity, respectively. To interpret the predictive contributions of individual features in the optimal model, SHAP was used. The analysis showed Leu as the most influential positive predictor of general and central obesity followed by Val and Ile, as shown in Figure 8. These findings indicate the relative importance of individual BCAAs for predicting obesity risk in adolescents and children.

## 4 Discussion

Using nationally representative data from CCLWNHS, this study comprehensively explored the associations of dietary BCAAs intake with risk of obesity, measured by both BMI and WC, among Chinese adolescents and children. Our results show that higher BCAAs intake is significantly related to higher prevalence of general and central obesity. These associations were robust after adjustment for several possible confounders and showed clear dose-response relationships. Furthermore, each of the three individual BCAAs, Ile, Leu, and Val, were each independently and positively associated with risk of obesity. Together, these results indicate that dietary BCAAs intake may be independently and positively associated with obesity risk in Chinese adolescents and children.

The inconsistent results on the relationship between dietary BCAAs intake and obesity in studies of adults may be explained by heterogeneity in the association between dietary intake and serum levels. Dietary intake of BCAAs does not necessarily indicate their true biological impact *in vivo*, while serum BCAAs levels are a direct indication of metabolic status, utilization efficiency, and metabolite accumulation, and represent a more accurate measure of the physiological impact (7). Numerous studies have shown consistent strong positive associations between serum BCAAs concentrations and overweight or obesity, making them reliable biomarkers and potential contributors to obesity and associated metabolic dysfunction in diverse populations and age groups. For example, studies of Chinese adults have demonstrated that increased serum levels of BCAAs are related to increased WC, BMI, body fat mass and visceral fat, and positively correlated with obesity risk (14, 38). Among newly diagnosed type 2 diabetes patients in Yunnan, China, Val, Leu, and Ile levels were significantly positively correlated with visceral adipose tissue, BMI, and BW (39). Similarly, one prospective study of Swiss adults noted positive correlations between serum BCAAs and anthropometric measures, such as BMI and WC (40), and US women with higher BMI had significantly higher serum levels of BCAAs (41). In adolescents and children, this association seems to be even more consistent and pronounced. A US cohort study displayed that serum BCAAs concentrations were positively related with insulin resistance and risk of obesity (42). Two other US studies found significantly higher serum BCAAs concentrations in obese vs. normal-weight adolescents (43, 44). Similarly, a Polish case-control study reported high levels of Leu, Ile, and Val in obese adolescents and children compared with normal-weight controls (45), and another cross-sectional survey of Polish prepubertal children reported similar findings (46). Additionally, a Brazilian study reported a positive correlation between serum BCAAs levels and BMI in children aged 7–12 years (47) and a Japanese study of obese children aged 9–10 years reported substantial positive correlations between serum Val, Leu, and Ile and BMI (48). To support a causal relationship, bariatric surgery in obese patients has been shown to significantly lower serum BCAAs levels, further linking BCAAs metabolism to obesity (49). Collectively, these findings suggest that increased serum BCAAs levels are a consistent and reliable biomarker of risk for obesity and obesity-related metabolic disorders, thus highlighting the important role of these peptides in the pathophysiology of obesity.

Although many studies have shown a positive correlation between dietary BCAAs intake and serum BCAAs levels (50–52), and restriction of dietary BCAAs has been shown to decrease serum fasting BCAAs concentrations (53, 54), some studies show no clear correlation (55). This heterogeneity may be a result of differences in the metabolic capacity of populations, the role of food matrices and general dietary patterns, and methodological limitations. As a result, dietary BCAAs intake may not fully represent *in vivo* serum levels, potentially “attenuating” or “shifting” observed associations with obesity and contributing to inconsistent findings across studies.

In the present study, we found a significant non-linear dose-response relationship between dietary BCAAs intake and the risk of overweight and obesity in adolescents and children, with the rate of risk increase slowing down after a certain threshold. This pattern may represent a saturation effect of serum BCAAs at higher intake levels (56), consistent with evidence from rodent studies (57), suggesting conserved physiological regulatory mechanisms of BCAAs metabolism across species.

Of particular note, the link between dietary BCAAs intake and central obesity was stronger for males, which is in agreement with prior studies (38, 39, 58, 59). This sex-specific difference might be due to bidirectional regulation by sex hormones: testosterone may have an enhancing effect on mammalian target of rapamycin complex 1 (mTORC1) signaling (60, 61), while estrogen promotes BCAAs clearance by activating the rate-limiting enzyme BCKDH, maintaining insulin sensitivity, and reducing lipogenesis (39). Differences in dietary sources may also play a role as males generally eat more animal-based proteins, which are better able to deliver BCAAs than plant proteins (62, 63). Excess BCAAs from these sources may be more readily converted to fat, which may increase the phenotypic association with obesity in males. In addition, the sex-specific observation could also be explained by inherent differences in body fat distribution between sexes. Males tend to develop a visceral/abdominal pattern of obesity, and females tend to develop a lower-body/gluteofemoral pattern of obesity. It has been demonstrated that lower-body adipose tissue has a reduced inflammatory profile and increased metabolic protective capacity, which is resistant to pro-inflammatory and hypometabolic changes in gene expression in the face of obesity. Conversely, insulin resistance and chronic low-grade inflammation are closely associated with android obesity, which is defined by the presence of abdominal fat (64–66). Consistently, a study using speckle-tracking echocardiography further confirmed this distinction at the level of cardiac function: android obesity, characterized by a higher waist-to-hip ratio, was independently associated with subclinical myocardial dysfunction, whereas gynoid obesity was associated with preserved or even superior myocardial function (67). These findings suggest that android obesity not only exhibits a worse metabolic profile but also confers greater susceptibility of target organs (e.g., the heart) to obesity-related adverse effects. Accordingly, the higher correlation of high BCAAs consumption with central obesity in males could be partly due to their higher vulnerability to the metabolically unfavorable visceral obesity phenotype. The excessive consumption of BCAAs can interact with the naturally pro-inflammatory and pro-insulin-resistant condition of android obesity, which together promotes the accumulation of fat in the central part of the body and related cardiometabolic risks.

Although Leu, Ile, and Val are all members of the BCAAs family, our research found that Leu has the strongest effect on fat metabolism and development of obesity. The physiological mechanisms through which Leu may play a role in obesity can be summarized in three important pathways. First, as a strong activator of the mTOR pathway, chronic excessive Leu intake causes overactivation of this signaling cascade, resulting in impaired insulin signaling, decreased peripheral glucose uptake, insulin resistance, and increased energy storage as fat (7, 68, 69). Concurrently, Leu increases lipogenic molecule expression, which increase triglyceride synthesis and lipid deposition (70, 71). Second, the long-term overconsumption of Leu may lead to persistent activation of hypothalamic mTOR signaling, which inhibits leptin receptor-mediated signaling. This decreases the hypothalamus sensitivity to circulating leptin (central leptin resistance), impairing satiety signals and enhancing feeding drive (72). Third, excessive Leu can affect the gut microbiota composition, leading to gut dysbiosis, imbalance of host energy metabolism, and indirectly favor fat accumulation (73).

Val and Ile also have different metabolic effects. Val catabolism produces 3-hydroxyisobutyrate (3-HIB) which favors lipid accumulation and insulin resistance (74, 75). 3-HIB can also induce chronic inflammation, which in turn favors fat accumulation. Moreover, Val may act on hepatocytes, upregulating lipogenic genes and worsening hepatic steatosis (7, 76). High Ile levels may impair the fibroblast growth factor 21 (FGF21) signaling, compromises brown adipose tissue thermogenesis and white adipose tissue “browning” to dissipate energy. Simultaneously, high Ile disrupts the balance between lipolysis and lipogenesis, enhancing triglyceride storage and fat accumulation (77). Collectively, the three BCAAs exert additive and synergistic effects to be related to excessive fat accumulation, obesity, and associated metabolic disorders. These results imply that holistic regulation of BCAAs metabolism may represent a potential target for obesity prevention and intervention strategy.

Based on available evidence, this is the first large-scale study to systematically investigate the relationship between dietary BCAAs intake and obesity among Chinese adolescents and children, and provides important exploratory insights. However, a number of limitations should be recognized. First, the cross-sectional design precludes causal inference regarding the association between BCAAs intake and obesity. Reverse causation is possible: children with higher adiposity may have dietary patterns that are richer in protein and thus BCAAs. The directionality of the association remains undetermined. Second, participants' food intake profiles were obtained by self-reported FFQ and 3-day weighed condiment inventory. Although these instruments were carefully validated, recall and measurement biases cannot be completely ruled out. Third, despite adjustment for multiple confounders, residual confounding may remain as a result of unmeasured or partially missing variables, including family socioeconomic status, parental obesity, and sleep quality. Additionally, although the sample contained four provinces with partial national representativeness, it did not represent all regions of China, which may limit generalizability. Finally, serum levels of BCAAs were not measured, so we were unable to validate dietary intake against metabolomic data, and assessment of *in vivo* BCAAs metabolic status was limited. As a result, the underlying causal mechanisms of the link between BCAAs intake and obesity are not fully understood. Future studies using prospective cohort designs, including a wider range of confounders, and combining metabolomic analyses are needed to elucidate the causality and biological mechanisms of BCAAs in childhood and adolescent obesity.

## 5 Conclusions

In conclusion, our study shows that a higher intake of dietary BCAAs is significantly related to higher risks of both central and general obesity in Chinese adolescents and children. Among the three BCAAs, Leu appeared to have the most prominent association with obesity risk, which may be a key contributor to the risk. These findings offer new evidence and suggest new targets for the development of precise, amino acid-based strategies to prevent and manage childhood obesity.

## Data Availability

The data analyzed in this study is subject to the following licenses/restrictions: The dataset used in this study is not publicly available due to ethical and privacy restrictions. Data are available from the National Institute of Nutrition and Health, Chinese Center for Disease Control and Prevention (China CDC) upon reasonable request and with permission from the Ethics Review Committee. Restrictions apply to the availability of these data, which were used under license for the current study. Requests for access to the dataset can be made to the National Institute of Nutrition and Health, Chinese Center for Disease Control and Prevention (China CDC) at http://www.chinanutri.cn/ or by contacting the corresponding author at lianlong00a@163.com.
