# Supplementary material for: Dietary branched-chain amino acids intake in relation to general and central obesity among Chinese children and adolescents: a cross-sectional study
Source: Front Nutr. 2026 May 21;13:1803824. doi: 10.3389/fnut.2026.1803824 (PMC13235303; doi:10.3389/fnut.2026.1803824)
Supplement: Supplementary file 1 [file Table_1.DOCX]

Supplementary Material

**Table S1**. ORs and corresponding 95% CIs according to dietary Ile intake and the risk of general obesity.

| **Variable** | **Classified Analysis** | | | | **Continuous Analysis** | |
| --- | --- | --- | --- | --- | --- | --- |
|  | **T1** | **T2** | **T3** | ***p* for trend** | **Per 1–SD** | ***p*–value** |
| General overweight |  |  |  |  |  |  |
| Case/control subjects, *n* | 477/2546 | 592/2546 | 616/2546 |  | 1685/7638 |  |
| Crude model | 1 | 1.24(1.09–1.42) | 1.29(1.13–1.47) | <0.001 | 1.30(1.14–1.47) | <0.001 |
| Model 1 | 1 | 1.26(1.11–1.44) | 1.32(1.15–1.50) | <0.001 | 1.31(1.15–1.48) | <0.001 |
| Model 2 | 1 | 1.22(1.05–1.41) | 1.22(1.02–1.46) | 0.039 | 1.23(1.01–1.46) | 0.040 |
| General obesity |  |  |  |  |  |  |
| Case/control subjects, *n* | 407/2546 | 544/2546 | 653/2546 |  | 1604/7638 |  |
| Crude model | 1 | 1.34(1.16–1.54) | 1.60(1.40–1.84) | <0.001 | 1.53(1.35–1.73) | <0.001 |
| Model 1 | 1 | 1.34(1.17–1.54) | 1.61(1.40–1.85) | <0.001 | 1.53(1.35–1.74) | <0.001 |
| Model 2 | 1 | 1.26(1.08–1.47) | 1.41(1.17–1.70) | <0.001 | 1.33(1.08–1.63) | 0.006 |
| General overweight and obesity |  |  |  |  |  |  |
| Case/control subjects, *n* | 884/2546 | 1136/2546 | 1269/2546 |  | 3289/7638 |  |
| Crude model | 1 | 1.29(1.16–1.43) | 1.44(1.30–1.59) | <0.001 | 1.41(1.28–1.56) | <0.001 |
| Model 1 | 1 | 1.30(1.17–1.44) | 1.45(1.31–1.60) | <0.001 | 1.41(1.28–1.56) | <0.001 |
| Model 2 | 1 | 1.23(1.10–1.39) | 1.30(1.13–1.50) | <0.001 | 1.27(1.09–1.49) | 0.003 |

Model 1 was adjusted for age (continuous), sex (female or male), MVPA (yes or no), school type (primary school or secondary school). Model 2 was further adjusted for protein (continuous), fat (continuous), carbohydrate (continuous). Abbreviations: SD: standard deviation.

**Table S2.** ORs and corresponding 95% CIs according to dietary Ile intake and the risk of central obesity.

| **Variable** | **Classified Analysis** | | | | **Continuous Analysis** | |
| --- | --- | --- | --- | --- | --- | --- |
|  | **T1** | **T2** | **T3** | ***p* for trend** | **Per 1–SD** | ***p*–value** |
| Pre-abdominal obesity |  |  |  |  |  |  |
| Case/control subjects, *n* | 641/2546 | 719/2546 | 743/2546 |  | 2100/7638 |  |
| Crude model | 1 | 1.12(1.00–1.26) | 1.15(1.03–1.30) | 0.019 | 1.15(1.02–1.29) | 0.019 |
| Model 1 | 1 | 1.13(1.00–1.28) | 1.16(1.03–1.31) | 0.016 | 1.15(1.03–1.29) | 0.016 |
| Model 2 | 1 | 1.13(0.99–1.30) | 1.16(0.98–1.37) | 0.083 | 1.17(0.97–1.40) | 0.095 |
| Abdominal obesity |  |  |  |  |  |  |
| Case/control subjects, *n* | 597/2546 | 791/2546 | 874/2546 |  | 2262/7638 |  |
| Crude model | 1 | 1.33(1.18–1.49) | 1.46(1.30–1.65) | <0.001 | 1.39(1.25–1.55) | <0.001 |
| Model 1 | 1 | 1.35(1.20–1.53) | 1.50(1.33–1.69) | <0.001 | 1.41(1.26–1.57) | <0.001 |
| Model 2 | 1 | 1.28(1.12–1.46) | 1.32(1.12–1.55) | 0.001 | 1.17(0.98–1.40) | 0.086 |
| Central obesity |  |  |  |  |  |  |
| Case/control subjects, *n* | 1238/2546 | 1510/2546 | 1614/2546 |  | 4362/7638 |  |
| Crude model | 1 | 1.22(1.11–1.34) | 1.30(1.19–1.43) | <0.001 | 1.27(1.16–1.39) | <0.001 |
| Model 1 | 1 | 1.24(1.13–1.36) | 1.32(1.21–1.45) | <0.001 | 1.28(1.17–1.40) | <0.001 |
| Model 2 | 1 | 1.20(1.08–1.33) | 1.23(1.08–1.40) | 0.002 | 1.17(1.01–1.35) | 0.036 |

Model 1 was adjusted for age (continuous), sex (female or male), MVPA (yes or no), school type (primary school or secondary school). Model 2 was further adjusted for protein (continuous), fat (continuous), carbohydrate (continuous). Abbreviations: SD: standard deviation.

**Table S3.** ORs and corresponding 95% CIs according to dietary Leu intake and the risk of general obesity.

| **Variable** | **Classified Analysis** | | | | **Continuous Analysis** | |
| --- | --- | --- | --- | --- | --- | --- |
|  | **T1** | **T2** | **T3** | ***p* for trend** | **Per 1–SD** | ***p*–value** |
| General overweight |  |  |  |  |  |  |
| Case/control subjects, *n* | 454/2546 | 600/2546 | 631/2546 |  | 1685/7638 |  |
| Crude model | 1 | 1.32(1.16–1.51) | 1.39(1.22–1.59) | <0.001 | 1.16(1.09–1.22) | <0.001 |
| Model 1 | 1 | 1.34(1.18–1.54) | 1.42(1.24–1.62) | <0.001 | 1.16(1.10–1.23) | <0.001 |
| Model 2 | 1 | 1.34(1.16–1.56) | 1.43(1.19–1.73) | <0.001 | 1.22(1.11–1.35) | <0.001 |
| General obesity |  |  |  |  |  |  |
| Case/control subjects, *n* | 373/2546 | 555/2546 | 676/2546 |  | 1604/7638 |  |
| Crude model | 1 | 1.49(1.29–1.72) | 1.81(1.58–2.08) | <0.001 | 1.29(1.22–1.37) | <0.001 |
| Model 1 | 1 | 1.50(1.30–1.73) | 1.81(1.57–2.08) | <0.001 | 1.29(1.22–1.37) | <0.001 |
| Model 2 | 1 | 1.47(1.26–1.73) | 1.77(1.45–2.15) | <0.001 | 1.37(1.24–1.52) | <0.001 |
| General overweight and obesity |  |  |  |  |  |  |
| Case/control subjects, *n* | 827/2546 | 1155/2546 | 1307/2546 |  | 3289/7638 |  |
| Crude model | 1 | 1.40(1.26–1.55) | 1.58(1.43–1.75) | <0.001 | 1.22(1.17–1.28) | <0.001 |
| Model 1 | 1 | 1.41(1.27–1.57) | 1.59(1.43–1.77) | <0.001 | 1.23(1.17–1.28) | <0.001 |
| Model 2 | 1 | 1.40(1.25–1.58) | 1.58(1.36–1.83) | <0.001 | 1.30(1.20–1.41) | <0.001 |

Model 1 was adjusted for age (continuous), sex (female or male), MVPA (yes or no), school type (primary school or secondary school). Model 2 was further adjusted for protein (continuous), fat (continuous), carbohydrate (continuous). Abbreviations: SD: standard deviation.

**Table S4.** ORs and corresponding 95% CIs according to dietary Leu intake and the risk of central obesity.

| **Variable** | **Classified Analysis** | | | | **Continuous Analysis** | |
| --- | --- | --- | --- | --- | --- | --- |
|  | **T1** | **T2** | **T3** | ***p* for trend** | **Per 1–SD** | ***p*–value** |
| Pre-abdominal obesity |  |  |  |  |  |  |
| Case/control subjects, *n* | 621/2546 | 747/2546 | 732/2546 |  | 2100/7638 |  |
| Crude model | 1 | 1.20(1.07–1.36) | 1.18(1.05–1.33) | 0.005 | 1.10(1.05–1.16) | <0.001 |
| Model 1 | 1 | 1.21(1.07–1.37) | 1.18(1.05–1.34) | 0.007 | 1.11(1.05–1.16) | <0.001 |
| Model 2 | 1 | 1.21(1.06–1.39) | 1.19(1.00–1.42) | 0.051 | 1.19(1.09–1.30) | <0.001 |
| Abdominal obesity |  |  |  |  |  |  |
| Case/control subjects, *n* | 566/2546 | 805/2546 | 891/2546 |  | 2262/7638 |  |
| Crude model | 1 | 1.42(1.26–1.61) | 1.57(1.40–1.77) | <0.001 | 1.23(1.17–1.29) | <0.001 |
| Model 1 | 1 | 1.45(1.28–1.63) | 1.61(1.43–1.81) | <0.001 | 1.24(1.17–1.30) | <0.001 |
| Model 2 | 1 | 1.40(1.22–1.60) | 1.52(1.28–1.80) | <0.001 | 1.28(1.17–1.39) | <0.001 |
| Central obesity |  |  |  |  |  |  |
| Case/control subjects, *n* | 1187/2546 | 1552/2546 | 1623/2546 |  | 4362/7638 |  |
| Crude model | 1 | 1.31(1.19–1.44) | 1.38(1.25–1.50) | <0.001 | 1.17(1.12–1.22) | <0.001 |
| Model 1 | 1 | 1.36(1.21–1.46) | 1.39(1.26–1.52) | <0.001 | 1.17(1.13–1.22) | <0.001 |
| Model 2 | 1 | 1.31(1.17–1.45) | 1.35(1.18–1.54) | <0.001 | 1.24(1.15–1.33) | <0.001 |

Model 1 was adjusted for age (continuous), sex (female or male), MVPA (yes or no), school type (primary school or secondary school). Model 2 was further adjusted for protein (continuous), fat (continuous), carbohydrate (continuous). Abbreviations: SD: standard deviation.

**Table S5.** ORs and corresponding 95% CIs according to dietary Val intake and the risk of general obesity.

| **Variable** | **Classified Analysis** | | | | **Continuous Analysis** | |
| --- | --- | --- | --- | --- | --- | --- |
|  | **T1** | **T2** | **T3** | ***p* for trend** | **Per 1–SD** | ***p*–value** |
| General overweight |  |  |  |  |  |  |
| Case/control subjects, *n* | 482/2546 | 581/2546 | 622/2546 |  | 1685/7638 |  |
| Crude model | 1 | 1.21(1.06–1.38) | 1.29(1.13–1.47) | <0.001 | 1.21(1.12–1.31) | <0.001 |
| Model 1 | 1 | 1.22(1.07–1.40) | 1.32(1.16–1.51) | <0.001 | 1.22(1.13–1.33) | <0.001 |
| Model 2 | 1 | 1.18(1.02–1.37) | 1.25(1.04–1.51) | 0.021 | 1.28(1.11–1.48) | 0.001 |
| General obesity |  |  |  |  |  |  |
| Case/control subjects, *n* | 379/2546 | 541/2546 | 684/2546 |  | 1604/7638 |  |
| Crude model | 1 | 1.43(1.24–1.65) | 1.80(1.57–2.07) | <0.001 | 1.42(1.30–1.54) | <0.001 |
| Model 1 | 1 | 1.43(1.24–1.65) | 1.81(1.58–2.08) | <0.001 | 1.42(1.31–1.54) | <0.001 |
| Model 2 | 1 | 1.42(1.21–1.67) | 1.79(1.47–2.18) | <0.001 | 1.50(1.30–1.74) | <0.001 |
| General overweight and obesity |  |  |  |  |  |  |
| Case/control subjects, *n* | 861/2546 | 1122/2546 | 1306/2546 |  | 3289/7638 |  |
| Crude model | 1 | 1.30(1.17–1.45) | 1.52(1.37–1.68) | <0.001 | 1.31(1.23–1.40) | <0.001 |
| Model 1 | 1 | 1.31(1.18–1.46) | 1.53(1.38–1.70) | <0.001 | 1.32(1.24–1.40) | <0.001 |
| Model 2 | 1 | 1.28(1.14–1.44) | 1.47(1.27–1.71) | <0.001 | 1.39(1.24–1.56) | <0.001 |

Model 1 was adjusted for age (continuous), sex (female or male), MVPA (yes or no), school type (primary school or secondary school). Model 2 was further adjusted for protein (continuous), fat (continuous), carbohydrate (continuous). Abbreviations: SD: standard deviation.

**Table S6.** ORs and corresponding 95% CIs according to dietary Val intake and the risk of central obesity.

| **Variable** | **Classified Analysis** | | | | **Continuous Analysis** | |
| --- | --- | --- | --- | --- | --- | --- |
|  | **T1** | **T2** | **T3** | ***p* for trend** | **Per 1–SD** | ***p*–value** |
| Pre-abdominal obesity |  |  |  |  |  |  |
| Case/control subjects, *n* | 637/2546 | 721/2546 | 742/2546 |  | 2100/7638 |  |
| Crude model | 1 | 1.13(1.01–1.28) | 1.17(1.03–1.31) | 0.013 | 1.14(1.05–1.22) | 0.001 |
| Model 1 | 1 | 1.14(1.01–1.29) | 1.17(1.04–1.32) | 0.011 | 1.14(1.06–1.23) | 0.001 |
| Model 2 | 1 | 1.14(1.00–1.31) | 1.16(0.98–1.38) | 0.087 | 1.23(1.08–1.41) | 0.002 |
| Abdominal obesity |  |  |  |  |  |  |
| Case/control subjects, *n* | 572/2546 | 773/2546 | 917/2546 |  | 2262/7638 |  |
| Crude model | 1 | 1.35(1.20–1.53) | 1.60(1.43–1.80) | <0.001 | 1.33(1.24–1.43) | <0.001 |
| Model 1 | 1 | 1.37(1.22–1.55) | 1.64(1.46–1.85) | <0.001 | 1.34(1.25–1.44) | <0.001 |
| Model 2 | 1 | 1.35(1.18–1.55) | 1.60(1.35–1.90) | <0.001 | 1.39(1.23–1.58) | <0.001 |
| Central obesity |  |  |  |  |  |  |
| Case/control subjects, *n* | 1209/2546 | 1494/2546 | 1659/2546 |  | 4362/7638 |  |
| Crude model | 1 | 1.24(1.13–1.36) | 1.37(1.25–1.51) | <0.001 | 1.23(1.16–1.31) | <0.001 |
| Model 1 | 1 | 1.25(1.14–1.38) | 1.40(1.27–1.53) | <0.001 | 1.24(1.17–1.32) | <0.001 |
| Model 2 | 1 | 1.24(1.12–1.38) | 1.36(1.19–1.56) | <0.001 | 1.32(1.19–1.46) | <0.001 |

Model 1 was adjusted for age (continuous), sex (female or male), MVPA (yes or no), school type (primary school or secondary school). Model 2 was further adjusted for protein (continuous), fat (continuous), carbohydrate (continuous). Abbreviations: SD: standard deviation.
